# Modeling Disease Trajectories for Castration-resistant Prostate Cancer Using Nationwide Population-based Data

**DOI:** 10.1016/j.euros.2022.07.010

**Published:** 2022-08-23

**Authors:** Eugenio Ventimiglia, Anna Bill-Axelson, Jan Adolfsson, Markus Aly, Martin Eklund, Marcus Westerberg, Pär Stattin, Hans Garmo

**Affiliations:** a Department of Surgical Sciences, Uppsala University, Uppsala, Sweden; b Division of Experimental Oncology/Unit of Urology, Urological Research Institute, IRCCS Ospedale San Raffaele, Milan, Italy; c Department of Clinical Science, Intervention and Technology, Karolinska Institutet, Stockholm, Sweden; d Department of Molecular Medicine and Surgery, Karolinska Institutet, Stockholm, Sweden; e Department of Pelvic Cancer, Karolinska University Hospital, Stockholm, Sweden; f Department of Medical Epidemiology and Biostatistics, Karolinska Institutet, Stockholm, Sweden

**Keywords:** Castration-resistant prostate cancer, Survival, State transition model

## Abstract

**Background:**

Little is known about disease trajectories for men with castration-resistant prostate cancer (CRPC).

**Objective:**

To create a state transition model that estimates time spent in the CRPC state and its outcomes.

**Design, setting, and participants:**

The model was generated using population-based prostate-specific antigen data from 40% of the Swedish male population, which were linked to nationwide population-based databases. We compared the observed and predicted cumulative incidence of transitions to and from the CRPC state.

**Outcome measurements and statistical analysis:**

We measured time spent in the CRPC state and the proportion of men who died of prostate cancer during follow-up by CRPC risk category.

**Results and limitations:**

Time spent in the CRPC state varied from 1.1 yr for the highest risk category to 3.9 yr for the lowest risk category. The proportion of men who died from prostate cancer within 10 yr ranged from 93% for the highest risk category to 54% for the lowest. There was good agreement between the model estimates and observed data.

**Conclusions:**

There is large variation in the time spent in the CRPC state, varying from 1 yr to 4 yr according to risk category.

**Patient summary:**

It is possible to accurately estimate the disease trajectory and duration for men with castration-resistant prostate cancer.

## Take Home Message

It is possible to accurately estimate disease trajectories and duration for men with castration-resistant prostate cancer.

## 1 Introduction

Estimates of long-term disease trajectories are essential in order to assess the impact of chronic diseases on health care systems. We recently reported a state transition model that can be used to provide estimates of the disease trajectory in men with prostate cancer (PCa) [1]. State transition models can overcome some of the limitations associated with the use of observational data, such as lack of long-term follow-up data and unregistered treatment changes. Furthermore, state transition models can take into account factors that affect clinical decisions, such as changes in comorbidities during follow-up [2]. A limitation of our model was that it did not include the castration-resistant PCa (CRPC) state, a pivotal determinant of disease trajectories in advanced-stage PCa [3]. Survival estimates for men with CRPC from recently published trials are available, although it is not clear if these estimates are applicable to the general population [4]. Moreover, no information regarding disease trajectories can be drawn from these trials and little is known regarding CRPC disease trajectories in current clinical practice. Therefore, we implemented the CRPC state in our model with the aim of estimating the duration of the CRPC state and outcomes for men in the CRPC state [5], [6].

## 2 Patients and methods

### 2.1 Study cohorts

We applied a state transition methodology [1] to the Uppsala-Örebro Prostate-specific antigen (PSA) Cohort (UPSAC) and the Stockholm PSA and Biopsy Register (STHLM-0), which together hold information on PSA testing for 40% of the male Swedish population [7], [8]. We then linked these cohorts to the National Prostate Cancer Register (NPCR) of Sweden and to several other national health care registers, including the Prescribed Drug Register, the Patient Register, and the Cause of Death Register, as previously done for the Prostate Cancer database of Sweden (PCBaSe) [9]. We then identified men with a PCa diagnosis who on January 1, 2006 or thereafter had filled prescriptions for gonadotropin-releasing hormone (GnRH) agonists and had PSA levels recorded. Treatment trajectories were identified by applying methods previously described for PCBaSe^Traject^ [9]. We started follow-up when each man had received 3 mo of GnRH treatment during a period of 6 mo or on the date of bilateral orchidectomy under the assumption that a man had reached castrate levels of testosterone at that time point, in other words, that he was in the castration-sensitive PCa (CSPC) state. Men were considered to be in the CSPC state during follow-up according to the same criteria. Men entered the CRPC state at the first date of doubling of their PSA nadir value with the last value being >2 ng/ml, or an absolute increase in PSA of ≥5 ng/ml, and had been on androgen deprivation therapy (ADT) for 3 mo or had been surgically castrated.

### 2.2 Statistical analyses

**Fig. 1:**
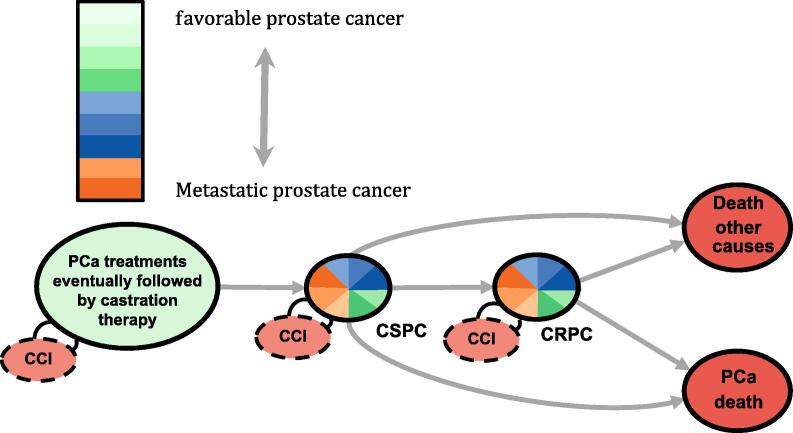
States and transitions for the proposed state transition model. Graphical representation of the state transition model to define transitions (arrows) between states (circles), including other prostate cancer (PCa) states, castration-sensitive prostate cancer (CSPC), castration-resistant prostate cancer (CRPC), death, and prostate cancer death. Multicolored circles represent transient stages and orange circles represent absorbing states. The small circles represent additional information gathered to facilitate estimation of the transition probabilities (Charlson Comorbidity Index [CCI]). The colors for transient states indicate disease severity categories at date of entry to the state.

The state transition model, including states, treatment trajectories, and transition probabilities to and from the CRPC state, is shown in Figure 1. The lack of data on testosterone levels in the study men was addressed by only allowing transitions to the CRPC state for men fulfilling the conditions for CSPC. Men in the CSPC state were categorized into eight risk categories according to PSA levels measured during the 3-mo period after initiation of GnRH (Supplementary Table 1). Men treated with orchidectomy entered the CSPC state on the date of surgery and the CSPC risk category remained the same as for the previous GnRH state described in the original state transition model [1]. Men remained in the CSPC state until either progression to CRPC or death. The date of CRPC was defined by an increase in PSA, either on the first date of the doubling of the nadir PSA value with the last value >2 ng/ml, or an absolute increase of ≥5 ng/ml, whichever event occurred first [8]. Men reaching the CRPC state were assigned a CRPC risk category on the basis of their PSA at CRPC and PSA doubling time (DT). It was recently shown that a linear combination of these PSA-derived measures is highly predictive of PCa death [10]. This “combined PSA kinetics risk” is calculated according to the equation:

$$log\left(PSA\;at\;CRPC\right)-1.4\times \;log\left(PSA\;DT\right).$$

We then created eight CRPC risk categories that we used in our model (Supplementary Table 2).

Transition probabilities for death from other causes were drawn from the original state transition model [1]. New transition probabilities were estimated for the following transitions: CSPC → CRPC, CSPC → PCa death, and CRPC → PCa death (Fig. 1). The probabilities of events within a 28-d period were modeled using logistic regression. Further information regarding these models is provided in the Supplementary material.

We then ran a microsimulation of every man in our set of data using a 28-d time interval until death or end of the simulated follow-up time (ie, 10 yr). To decrease random errors, we ran the simulation 100 times for each man. We then extracted and compared the observed and predicted cumulative incidence of transitions to the CSPC state, the CRPC state, and death from PCa. We then compared the cumulative incidence of state transitions between simulated and observed data and we cross-validated the models by applying the model from the UPSAC cohort to the STHLM-0 cohort and vice versa. Finally, we estimated the time spent in the CRPC state as well as the proportion of men who died of PCa during follow-up according to CRPC risk categories.

## 3 Results

To build the state transition model, we identified two cohorts: (1) men from STHLM-0 or UPSAC who reached the CSPC state (*n* = 7263 men) and (2) men who transitioned to the CRPC state (*n* = 3899 men).

**Fig. 2:**
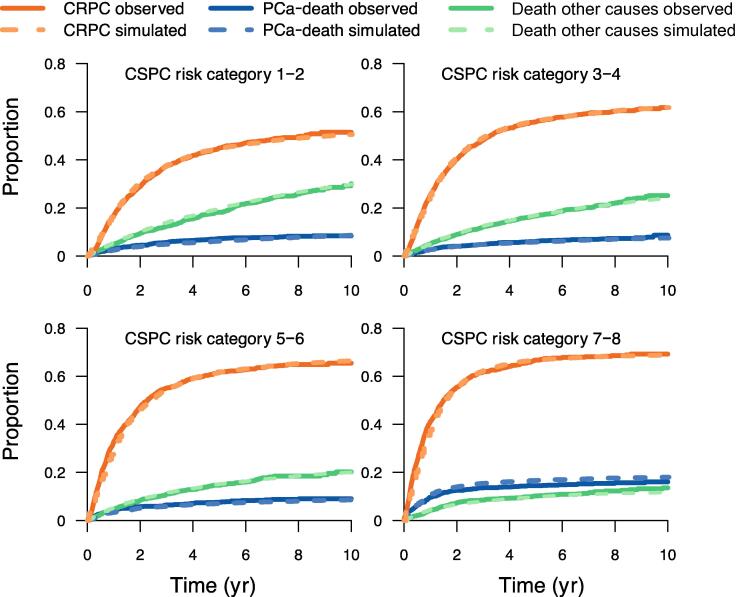
Cumulative incidence of transition to the castration-resistant prostate cancer (CRPC) state for men in the castration-sensitive prostate cancer (CSPC) state according to risk category.

Characteristics of these cohorts are shown in Table 1. Approximately 60% of men were older than 75 yr, and more than 60% received GnRH as their primary treatment. Moreover, more than 60% of the men had no comorbidities (Charlson Comorbidity Index = 0). Risk categories were skewed towards the lower end among men in the CSPC state, whereas the risk categories were more evenly distributed among men in the CRPC state. Transition to CRPC was much more common and rapid for men in the highest CSPC risk category (55% at 2 yr) compared to the lowest risk category (30% at 2 yr; Fig. 2).

**Table 1:** Baseline characteristics for men in the CSPC and CRPC states

	CSPC (*n* = 7263)	CRPC (*n* = 3899)
Age at state entry, *n* (%)		
≤65 yr	809 (11.1)	432 (11.1)
≤66–75 yr	2196 (30.2)	1192 (30.6)
76–85 yr	3230 (44.5)	1629 (41.8)
≥86 yr	1028 (14.2)	646 (16.6)
Year of diagnosis, *n* (%)		
<2006	1173 (16.2)	630 (16.2)
2006–2008	1860 (25.6)	1108 (28.4)
2009–2011	2063 (28.4)	1277 (32.8)
2012–2014	2167 (29.8)	884 (22.7)
Time from diagnosis to ADT, *n* (%)		
≤1 yr	4537 (62.5)	847 (21.7)
1–4 yr	1147 (15.8)	1858 (47.7)
≥4 yr	1579 (21.7)	1192 (30.6)
Charlson Comorbidity Index, *n* (%)		
0	4556 (62.7)	2398 (61.5)
1	1174 (16.2)	630 (16.2)
2	647 (8.9)	375 (9.6)
≥3	886 (12.2)	496 (12.7)
Treatment history, *n* (%)^a^		
Primary GnRH	4543 (62.5)	2556 (65.6)
AA → GnRH	683 (9.4)	463 (11.9)
WW → AA → GnRH	204 (2.8)	102 (2.6)
WW → GnRH	885 (12.2)	328 (8.4)
RP → AA → GnRH	162 (2.2)	82 (2.1)
RT → AA → GnRH	142 (2.0)	90 (2.3)
RP → GnRH	208 (2.9)	86 (2.2)
RT → GnRH	436 (6.0)	192 (4.9)
CSPC/CRPC risk category, *n* (%)		
1–2	2119 (29.2)	849 (21.8)
3–4	2715 (37.4)	1147 (29.4)
5–6	1329 (18.3)	963 (24.7)
7–8	1100 (15.1)	940 (24.1)

AA = antiandrogen; ADT = androgen deprivation therapy; CSPC = castration-sensitive prostate cancer; CRPC = castration-resistant prostate cancer; GnRH = gonadotropin-releasing hormone agonist; RP = radical prostatectomy; RT = radiotherapy.

a Excluding the CSPC state for men in the CRPC state.

**Fig. 3:**
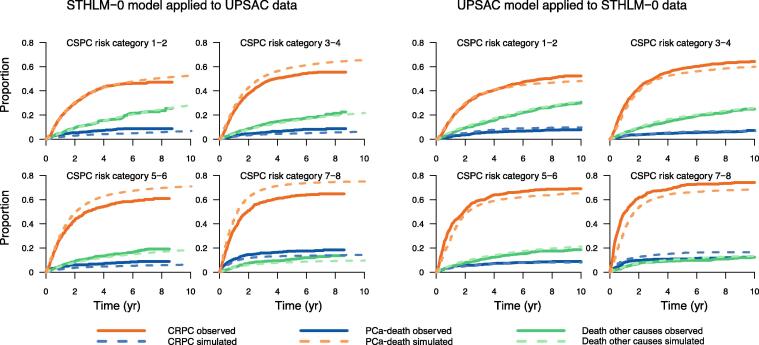
Cross-validation of the cumulative incidence of transition to the castration-resistant prostate cancer (CRPC) state for men in the castration-sensitive prostate cancer (CSPC) state.UPSAC = Uppsala-Örebro Prostate-specific antigen (PSA) Cohort; STHLM-0 = Stockholm PSA and Biopsy Register.

To test the validity of our model, we compared the cumulative incidence of observed and predicted transitions. Overall, there was good agreement between observed and predicted transitions for all risk categories, both for cumulative incidence of transition to CRPC (Fig. 3) and for PCa death (Supplementary Fig. 1).

The predicted time spent in the CRPC state during the first 10 yr following castration varied from 1.1 yr for the highest risk category to 3.9 yr for the lowest risk category. The proportion of men who died from PCa within 10 yr ranged from 93% for the highest risk category to 54% for the lowest risk category. These estimates are in good agreement with observed data, confirming that the internal validity of our model was good (Supplementary Table 3).

## 4 Discussion

In our state transition model of men with CRPC there was a large range for the duration of the CRPC state according to risk categories based on PSA-derived measures. Men with CRPC in the highest risk category spent only 1 yr in the CRPC state, whereas men in the lowest risk category spent 4 yr in the CRPC state. Correspondingly, 93% of men in the highest risk category and 54% of men in the lowest risk category had died 10 yr after transition to the CRPC state.

One of the advantages of our study is that we based our state transition model on a cohort capturing 40% of the Swedish male population, so it is highly representative of the general population. Moreover, the use of high-quality data from national health care registers allowed us to track disease trajectories as well as outcomes. There are some limitations to our study. We addressed the lack of data on testosterone levels by introducing and estimating the CSPC state, which was only possible to reach if PSA measurement happened while the patient was considered exposed to GnRH. Further limitations include the possibility of regional differences between the two cohorts (STHML-0 and UPSAC) in terms of pattern of PSA measurements. The lack of data on the presence of metastases is also a limitation, since men with nonmetastatic CRPC have longer survival than men with metastatic CRPC [11]. Although the PSA level at transition to CRPC may represent a proxy for both tumor volume and metastatic status, future inclusion of results on imaging of bone and parenchyma will improve the model [12]. Another limitation is the lack of information regarding chemotherapy, which is not usually recorded in the Prescribed Drug Register [13]. Furthermore, it is not yet possible to obtain errors from the statistical estimations in our model; a refined version of the model will be implemented in the future in order to fill this gap.

Despite the interest in CRPC owing to the advent of novel drugs for this disease state, there is little information on time spent in the castration-resistant state in clinical practice. The survival observed in our study, ranging from 1 to 4 yr, depending on the risk category, is in line with a previous report based on clinical practice data from The Netherlands [4]. In randomized clinical trials, men with metastatic CRPC had median survival of approximately 3.5 yr [14]. Men enrolled in trials are generally younger and have less comorbidity in comparison to men treated in routine clinical practice [15]. It should be noted that our study period was before the introduction of several novel treatments for CRPC that have been shown to increase survival. We used a combination of PSA at transition to CRPC and PSA DT to model the risk of death for men with CRPC. Higher PSA at the date of CRPC and shorter PSA DT were both associated with shorter survival [16], [17], [18]. We argue that the combination of PSA at the date of CRPC and PSA DT results in a clear proxy for disease aggressiveness, as shown by the different outcomes for men according to risk category.

To the best of our knowledge, this is the first report on disease trajectories using a population-based cohort of men on ADT who transition to CRPC that includes the time spent in the CRPC state. The comprehensive follow-up and the high-quality data from the population-based registers used in the study [19] represent major strengths. An increase in data granularity in terms of disease characteristics, together with the inclusion of more recently diagnosed men, is likely to result in more accurate estimates.

## 5 Conclusions

It is possible to estimate disease trajectories and key outcomes for CRPC using the updated version of our previously published state transition model, which provides accurate data on CRPC duration and survival outcome at a population-based level. Time spent in the CRPC state varies widely from 1 to 4 yr according to risk category.

  ***Author contributions***: Eugenio Ventimiglia had full access to all the data in the study and takes responsibility for the integrity of the data and the accuracy of the data analysis.

*Study concept and design*: Ventimiglia, Garmo, Stattin.

*Acquisition of data*: Bill-Axelson, Aly, Eklund.

*Analysis and interpretation of data*: Ventimiglia, Garmo.

*Drafting of the manuscript*: Ventimiglia.

*Critical revision of the manuscript for important intellectual content*: Stattin, Bill-Axelson, Adolfsson, Aly, Eklund, Westerberg.

*Statistical analysis*: Garmo.

*Obtaining funding*: Stattin.

*Administrative, technical, or material support*: Stattin, Bill-Axelson, Aly, Eklund.

*Supervision*: Stattin, Bill-Axelson, Adolfsson, Aly, Eklund.

*Other*: None.

  ***Financial disclosures:*** Eugenio Ventimiglia certifies that all conflicts of interest, including specific financial interests and relationships and affiliations relevant to the subject matter or materials discussed in the manuscript (eg, employment/affiliation, grants or funding, consultancies, honoraria, stock ownership or options, expert testimony, royalties, or patents filed, received, or pending), are the following: None.

  ***Funding/Support and role of the sponsor:*** The Swedish Cancer Society 19 0030 Pj 01 H.
