# Supplementary material for: Modeling Disease Trajectories for Castration-resistant Prostate Cancer Using Nationwide Population-based Data
Source: Eur Urol Open Sci. 2022 Aug 23;44:46–51. doi: 10.1016/j.euros.2022.07.010 (PMC9520495; doi:10.1016/j.euros.2022.07.010)
Supplement: Supplementary data 1 [file mmc1.docx]

**1. Transitions probabilities**

**1.1 Transition to castration state (CP)**

This state can be reached only from GnRH state. This transition was modelled in a logistic regression involving time spent in the GnRH state, age (modelled as a constant term below age 65 and linear above age 65), age (modelled as a constant term below age 85 and linear above age 85), CP risk categories, and previous history of treatment.

**1.2 Transition to castration resistant prostate cancer (CRPC)**

This state can be reached only from CP state. This transition was modelled in a logistic regression involving age, Charlson comorbidity index (0 vs 1 vs 2 vs 3 vs >3), time spent in the CP state, history of previous treatment, CP risk groups and GnRH risk groups.

**1.3 Transition to death from prostate cancer (DP)**

It is now possible to reach this final absorbing state also from CP and CRCP status. The transition CP 🡪 DP was modelled in a logistic regression involving age (modelled as a constant term below age 80 and as linear term above), Charlson comorbidity index (0 vs 1 vs 2 vs 3 vs >3), history of previous treatment, time spent in CP state, CP and GnRH risk categories. The transition CRPC 🡪 DP was modelled in a logistic regression involving age (modelled as a constant term below age 70 and as a linear term above), age (modelled as a constant term below age 90 and as a linear term above), Charlson comorbidity index (0 vs 1 vs 2 vs 3 vs >3), time spent in CRPC state, GnRH risk groups and CRPC risk groups.

**Supplementary Table 1. Risk categories for men on gonadotropin releasing hormone agonists (GnRH) who reach the castration status based on PSA-levels measured after initiation of GnRH**

| **Risk category** | **PSA after ADT initiation (ng/ml)** |
| --- | --- |
| CSPC_1_ | PSA<0.2 |
| CSPC _2_ | 0.2≤PSA<1 |
| CSPC _3_ | 1≤PSA<3 |
| CSPC _4_ | 3≤PSA<8 |
| CSPC _5_ | 8≤PSA<16 |
| CSPC _6_ | 16≤PSA<32 |
| CSPC _7_ | 32≤PSA<100 |
| CSPC _8_ | PSA≥100 |

ADT: androgen deprivation therapy

CSPC: castration sensitive prostate cancer

**Supplementary Table 2. Castration resistant prostate cancer risk categories.**

| **Risk category** | **PSA kinetics risk** |
| --- | --- |
| CRPC_1_ | *Combined PSA kinetics Risk* < -7 |
| CRPC_2_ | -7≤ *Combined PSA kinetics Risk* <-6 |
| CRPC_3_ | -6≤ *Combined PSA kinetics Risk* <-5 |
| CRPC_4_ | -5≤ *Combined PSA kinetics Risk* <-4 |
| CRPC_5_ | -4≤ *Combined PSA kinetics Risk* <-3 |
| CRPC_6_ | -3≤ *Combined PSA kinetics Risk* <-2 |
| CRPC_7_ | -2≤ *Combined PSA kinetics Risk* <-1 |
| CRPC_8_ | *Combined PSA kinetics Risk* ≥-1 |

CRPC: castration resistant prostate cancer

**Supplementary Table 3. Predicted and observed estimates of time spent in castration resistant prostate cancer (CRPC) state and proportion of men dying from prostate cancer (PCa) at the end of follow-up time according to different CRPC risk categories.**

| **Risk category** | **Time in CRPC (years)**  **Observed vs Predicted** | **Proportion of PCa death**  **Observed – Predicted** |
| --- | --- | --- |
| CRPC_1-2_ | 3.84 vs 3.95 | 53% vs 54% |
| CRPC_3-4_ | 3.10 vs 3.14 | 68% vs 71% |
| CRPC_5-6_ | 2.07 vs 2.10 | 83% vs 84% |
| CRPC_7-8_ | 1.04 vs 1.11 | 87% vs 93% |

**Supplementary Figure 1. Cross validation of cumulative incidence of death for men in the castration resistant prostate cancer state (CRPC).**
